# Impact of mass distribution of free long-lasting insecticidal nets on childhood malaria morbidity: The Togo National Integrated Child Health Campaign

**DOI:** 10.1186/1475-2875-9-199

**Published:** 2010-07-12

**Authors:** Dianne J Terlouw, Kodjo Morgah, Adam Wolkon, Aboudou Dare, Ameyo Dorkenoo, M James Eliades, Jodi Vanden Eng, Yao K Sodahlon, Feiko O ter Kuile, William A Hawley

**Affiliations:** 1 Child and Reproductive Health, Liverpool School of Tropical Medicine, Pembroke Place, Liverpool, UK; 2 National Malaria Control Programme, Togo Ministry of Health, PB 518, Lome, Togo; 3 Malaria Branch, Division of Parasitic Diseases, Centers for Disease Control and Prevention, 4770 Buford Highway NE, Atlanta, USA; 4 Child Survival and Development Cluster, UNICEF, Indonesia

## Abstract

**Background:**

An evaluation of the short-term impact on childhood malaria morbidity of mass distribution of free long-lasting insecticidal nets (LLINs) to households with children aged 9-59 months as part of the Togo National Integrated Child Health Campaign.

**Methods:**

The prevalence of anaemia and malaria in children aged zero to 59 months was measured during two cross-sectional household cluster-sample surveys conducted during the peak malaria transmission, three months before (Sept 2004, n = 2521) and nine months after the campaign (Sept 2005, n = 2813) in three districts representative of Togo's three epidemiological malaria transmission regions: southern tropical coastal plains (Yoto), central fertile highlands (Ogou) and northern semi-arid savannah (Tone).

**Results:**

In households with children <5 years of age, insecticide-treated net (ITN) ownership increased from <1% to >65% in all 3 districts. Reported ITN use by children during the previous night was 35.9%, 43.8% and 80.6% in Yoto, Ogou and Tone, respectively. Rainfall patterns were comparable in both years. The overall prevalence of moderate to severe anaemia (Hb < 8.0 g/dL) was reduced by 28% (prevalence ratio [PR] 0.72, 95% CI 0.62-0.84) and mean haemoglobin was increased by 0.35 g/dL (95% CI 0.25-0.45).

The effect was predominantly seen in children aged 18-59 months and in the two southern districts: PR (95% CI) for moderate to severe anaemia and clinical malaria: Yoto 0.62 (0.44-0.88) and 0.49 (0.35-0.75); Ogou 0.54 (0.37-0.79) and 0.85 (0.57-1.27), respectively. Similar reductions occurred in children <18 months in Ogou, but not in Yoto. No effect was seen in the semi-arid northern district despite a high malaria burden and ITN coverage.

**Conclusions:**

A marked reduction in childhood malaria associated morbidity was observed in the year following mass distribution of free LLINs in two of the three districts in Togo. Sub-national level impact evaluations will contribute to a better understanding of the impact of expanding national malaria control efforts.

## Background

Insecticide-treated nets (ITNs) have been shown to cause marked reductions in all-cause child mortality, a 50%, 29% and 13% reduction in uncomplicated malaria, high density parasitaemia and any parasitaemia, respectively, and an increase in average haemoglobin level by 1.7% packed cell volume, in children under five years of age in areas of stable transmission [1]. As the value of ITNs as a malaria control tool has been convincingly demonstrated in study settings, efforts have shifted towards implementation and rapid scale-up of coverage at country level. One delivery strategy that has received considerable attention is the mass distribution of free ITNs linked to established vaccination campaigns, an approach pioneered by the Red Cross and the AMP (Alliance for Malaria Prevention). Following two smaller scale pilot projects in Ghana and Zambia [2,3], Togo carried out the first ever integrated national scale distribution of long-lasting insecticidal nets (LLINs) linked to a measles immunization campaign in December 2004 [4]. This campaign aimed to lower morbidity and mortality in young children in Togo through a combination of four interventions; measles vaccination, polio vaccination, LLINs, and presumptive treatment for helminth infection. The target group of this campaign was determined by the measles vaccination component, and included children aged nine to 59 months. However, the distributed LLINs were large and could cover more than one individual, and the campaign had the potential to directly protect approximately 25% of the population of Togo.

Evaluation of short-term programme impact is a core part of current efforts to rapidly scale-up malaria control efforts, but poses real challenges [5]. The indicators to estimate accurately the malaria disease burden at population level via household surveys are limited and not malaria specific. Furthermore, as randomly selected contemporaneous control groups without nets are no longer available, impact evaluations rely on the assessment of trends in disease burden over time. With this, the strength of inference about the causality of the observed effect becomes a plausibility rather than a probability argument--i.e. one makes an assumption that observed reductions in morbidity can be attributed to the control efforts if the population-level coverage of malaria control interventions has increased, as one cannot rule out alternative explanations [6]. Because of natural variation in geographical, seasonal and annual malaria transmission, repeated cross-sectional household level impact evaluations (every 3-4 years) are recommended to help establish trends over time [7,8]. To achieve a relatively high level of plausibility in this short-term pre-post impact assessment, it was controlled for known confounding factors, the presence of an 'intensity of exposure-response' effect in the post-intervention survey was assessed by comparing ITN users with non-users, and it was assessed whether the observed impact was compatible to the known efficacy of ITNs [6].

As the first example of rapid national scale up of ITNs using an integrated child health campaign, the Togo experience has been the subject of extensive evaluation. This impact evaluation was part of a multi-disciplinary monitoring and evaluation effort of the Togo campaign and included evaluation of ITN coverage, childhood morbidity, economic costing and the role of social mobilization. This paper assesses the impact of the campaign on malaria-associated morbidity, and consists of a set of two repeated prevalence surveys in children below five years of age. The results of the baseline morbidity survey have been presented previously [9], and indicated a high malaria burden, particularly in the first 1.5 to 2 years of life [9]. Findings from a coverage survey conducted one month after the campaign found that national household ownership of at least one ITN had increased from 8.0% to 62.5% [4]. The campaign-interventions were received by >90% of eligible children, with similar levels across the different socio-economic strata.

In this paper, results are presented of the September 2005 morbidity survey, conducted nine months after the campaign, and compared with the baseline results from 2004 to assess impact of the campaign on malaria morbidity in children below five years of age.

## Methods

**Figure 1:**
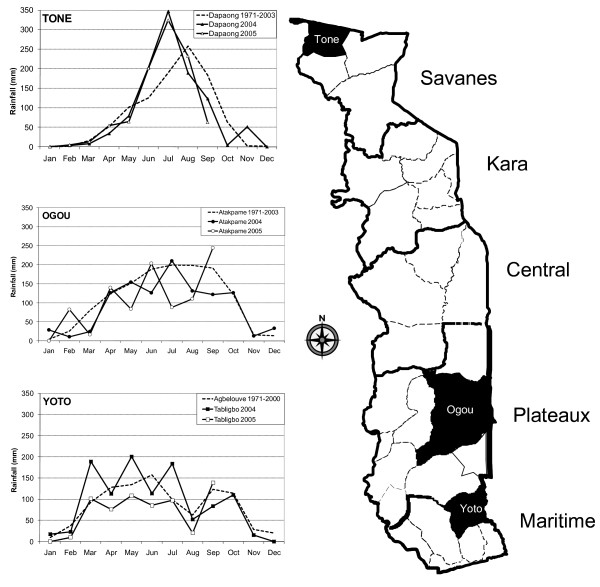
**Monthly rainfall in three districts of Togo for 2004 and 2005, with 30 year means for comparison**. Data are presented for the capital cities of each surveyed district.

A detailed description of the study sites, population, survey design and methodology has been published [9]. Briefly, the study was conducted in rural communities from three districts: Yoto in the tropical coastal climate of the southern Maritime Region, Ogou in the hilly, fertile central Plateaux Region (altitudes around 500 m), and Tone, the most northern district in the more semi-arid, Savanes Region that borders Burkina Faso. Malaria transmission in Togo reflects its rainfall and geographical patterns, with a bimodal pattern in the South that gradually changes into a single rainy season that progressively shortens to the North (Figure 1).

The methods, planning and implementation of the campaign have been previously described by Takpa *et al *[4]. During this two-week campaign, over 905,000 LLINs were distributed from 1,339 health posts. Households with more than one eligible child received either one or several nets, depending on the health post and other factors.

### Study population

Togo is 435 km long and 100 km across at its widest point and had a population of around 5.7 million in 2002. Approximately 17.2% or 980,000 children are under the age of five years [10] and there are an estimated 245,000 live births per year [11,12]. Most people in rural areas are subsistence farmers growing food crops of cassava, yams, corn, and rice. A few cash crops such as coffee, cacao, and cotton for export are also grown, mainly in the central Plateaux Region. Around 53% of the adult population in the country is literate, with the lowest literacy rate found in the northern Savanes Region [12].

### Study design and sample size

Two repeated community-based cross-sectional surveys were conducted involving children <5 years in Sept 2004 (three months pre-campaign) and Sept 2005 (nine months post-campaign). Both surveys were conducted during the estimated peak malaria transmission season (based on rainfall patterns and Ministry of Health (MoH) surveillance statistics) and used an identical design, with the difference in prevalence of moderate-to-severe anaemia (haemoglobin level (Hb) <8.0 g/dL) in children aged 0-59 months between surveys as the main endpoint. Haemoglobin levels were chosen as the primary outcome measure as they are sensitive to changes in malaria transmission, but have the valuable property of averaging out changing effects over time, and have been recommended by the RBM Malaria Monitoring and Evaluation Reference Group (MERG) [13-15]. As the campaign only targeted children between nine to 59 months, and the post-intervention survey occurred nine months post-campaign, this indicator included children <18 months who had not been targeted by the campaign, but who may have been sharing a campaign ITN with an older sibling in the household who had been.

The study was designed to allow evaluation of impact at district level and took into account the possible presence of effect modification by study region or age. A stratified two-stage cluster sample design was used. After selection of three geographically representative districts, a total of 90 enumeration areas were selected (30 per district) by random sampling with a probability proportional to size (PPS). Within each enumeration area all households were mapped using PDA-based GPS technology. A random sample of 25 (2004) or 27 (2005) households was selected regardless of the presence of children under the age of five years. Final clusters of enrolled households thus varied per EA. Selected households were revisited and invited to participate in the survey if there were children aged 0-59 months in the household, at which time the presence and hanging of an ITN was assessed. The morbidity survey took place two days later in a central location of each selected EA. The fieldwork for each of the two surveys was conducted during a three-week period. Six PDA teams of two staff members and six clinical survey teams consisting of five people each completed the survey in six enumeration areas per day. The use of PDA based GPS technology to determine a sampling frame in this study has been described in detail in a separate publication [16].

The study had an 80% power to detect a 33% difference between pre- and post-intervention prevalence of moderate-to-severe anaemia in children 0-59 months in each of the three districts with 95% confidence, assuming a baseline prevalence of 20%, and taking into account a 10% refusal rate, and a design effect of 1.5. The pooled sample size of 2,700 children per survey had a power of >85% to detect a 20% difference between surveys.

### Study procedures

Refusal and non-response was recorded at household level in 2004. In 2005 this was changed to collection at child level for practical reasons. The 2004 household level data was converted into child level data, assuming an average of 1.45 children per household in 2004. For each household, a structured questionnaire was completed with the caretaker of the child to record background data on demographics, socio-economic status and the child's reported medical history. The used questionnaire was an adapted version of the MIS questionnaire. Axillary temperature was measured twice during the questionnaire process. A capillary blood sample was taken to assess haemoglobin level (Hemocue, Angelholm, Sweden), the presence of malaria parasites were assessed by rapid diagnostic test (RDT, Paracheck Pf, Orchid Biomedical Systems, India), as well as a thin and thick malaria blood smear. Lastly, a brief medical exam was performed. Appropriate treatment was provided to children with anaemia (iron syrup 2-3 mg/kg/day of elementary iron for a minimum of four weeks) and parasitaemia as determined by RDT (artesunate-amodiaquine, provided by Sanofi-Synthelabo, France in 2004; and DAFRA, Belgium in 2005). Children with an acute illness that required further attention were referred to the nearest health facility. Malaria thick and thin smears were stained in the field with Giemsa (using a 3% Giemsa solution, stained for 30-45 minutes) and read at a later date at the malaria reference laboratory of the Ministry of Health in the capital Lome. The total number of asexual parasites and gametocytes was determined per 500 white blood cells, assuming a total white blood cell count of 8,000/μl. A subset of slides were reread by a second microscopist for quality assurance purposes. Monthly rainfall data was provided by the National Meteorological Service in Lome.

### Data management and statistical methods

Data were entered directly into a database using PDAs in the field (questionnaire application software: Visual CE 8.0, Syware Inc, Cambridge, Massachusetts) and downloaded into a central database (Microsoft Access). Data were entered and cleaned using range and internal consistency checks. Analyses were performed using SAS (version 9.1, SAS Institute, Cary, NC). Analyses were weighed for the number of invited households as this varied over the first 3 days of the 2004 survey. The overall estimated effect of LLINs in Togo was based on pooled district estimates, weighted for the proportion of the population that the three districts represented, (coastal plains, central highlands, and semi-arid savannah, representing 41.2%, 33% and 25.8% of the population, respectively based on the most recent DHS data). Comparisons between pre- and post-campaign survey were made on the basis of intention-to-treat (i.e. independent of reported ITN use in 2005). Crude weighted frequencies, percentages, and Rao-Scott chi-square tests of association were calculated using PROC SURVEYFREQ. Dichotomous outcomes of interest were assessed while controlling for other covariates using log-binomial regression with the PROC GENMOD procedure. Adjusted prevalences were obtained from least squares mean estimates using the LSMEANS output group in this procedure. The village based cluster randomization was taken into account using an exchangeable correlation structure for observations obtained from residents within one village.

A secondary analysis comparing current ITN users versus non-users in the 2005 survey used similar methods.

Linear regression models (PROC MIXED procedure) were used to determine the mean difference in continuous endpoints (e.g. haemoglobin level) between the pre- and post campaign survey using a robust variance estimation correcting for village based cluster randomization, and assessing the same co-variates as was done in the models for dichotomous endpoints. The design effect associated with the primary endpoint (the ratio of the cluster-corrected standard errors and the standard error computed under the assumption of a simple random sample design) was assessed using an alternating logistic regression model (ALR) with the PROC GENMOD procedure. The actual observed design effect at village level for moderate to severe anaemia was 1.07 (95%CI 1.01-1.15).

Age was included in each model. Because of the rapid physiological changes in haemoglobin levels in childhood, narrow age categories were used (0-5, 6-11, 12-17, 18-23, 24-35, 36-47, 48-59 months). The presence of effect modification by district and age of the association between survey and various outcomes was assessed by adding a survey × district × age interaction term in the model, with age as a binary (0-17 and 18+ months) variable, and was determined by the P-value of the interaction term (using P < 0.05 as significant). As there was evidence of effect modification by district × age (as a binary variable 0-17 and 18+ months) of the impact of LLINs on moderately severe anaemia and clinical malaria, results are also presented separately for subgroups. Other variables considered for inclusion were: sex of the child, household socio-economic status, and level of education. If covariates were significant determinants of the outcome evaluated, but not confounders of the relationships of interest (i.e. pre- and post-intervention survey and outcome), and had no or minimal impact on the precision of the point estimates, they were excluded from the final models. For all statistical tests a two-sided *P*-value < 0.05 was considered significant.

## Definitions

A household was defined as a unit of one caretaker and her children. In polygynous households, one woman was randomly selected and invited to participate with her children <5 years. Socio-economic status was defined in quintiles according to a wealth index score based on criteria developed by the World Bank to classify each household's economic status [17]. Malaria indicators were selected in line with globally recommended RBM indicators. Anaemia, moderate-to-severe anaemia, and severe anaemia were defined as haemoglobin concentrations <11.0 g/dL, <8.0 g/dL and <5.0 g/dL, respectively. Malaria parasitaemia was defined as any asexual parasitaemia of any species detected on a thick or thin blood smear. High density parasitaemia was defined as any asexual parasitaemia ≥5,000/μL. Clinical malaria was defined as a documented axillary temperature ≥37.5°C in the presence of any asexual malaria parasitaemia based on the slide result. Long-Lasting Insecticidal Nets (LLINs) are defines as ready-to-use pretreated mosquito nets, which require no further treatment during its expected life span (average 4 to 5 years). Insecticide Treated Nets are defined as untreated nets which have been impregnated with insecticide and need to be retreated every 6-12 months. In this paper we use ITNs to refer to a combination of the distributed campaign LLINs and other ITNs. "ITN use" was defined as a positive answer at the time of the morbidity survey in a central location to the question 'did this person sleep under an ITN last night?', while "ITN hanging" was defined as a positive answer during the home visit from the GPS team to the question whether a net was hanging. At this time, if allowed, staff visually confirmed that an ITN was tied over a sleeping space, even if the net was folded above the bed. Nets that were out of the package and not hanging, but were reported to have been hanging the previous night were reported as hanging. Nets not tied above a sleeping space, reported not used the previous night or still in their packaging were categorized as 'not hanging'.

## Results

In total, 2,304 and 2,430 households were randomly selected for participation in the 2004 and 2005 surveys, respectively. These households reported the presence of 2,721 and 3,123 children below the age of five years in 2004 and 2005, who were invited to participate in the survey. Of the invited children 70 and 42 were subsequently excluded because their dates of births revealed they were 60 months or older at the time of the survey. Dates of births were recorded from their under-5 clinic cards where available. Of the eligible children, 95.5% (2,532/2,651) and 96.3% (2,967/3,081) were enrolled. The main reason for non-enrolment was not being at home due to travel or work (4.4% and 3.4%). Only 3 (0.1%) and 9 (0.3%) children were not enrolled due to a refusal by their caretakers. A further 165 records were excluded from the final analyses because a) children were erroneously enrolled as part of a second or third mother-children pair within households of polygynous families in 2005 in Tone (147 records) or, b) because of data quality problems (18 records). In total, 5,334 children contributed to the results; 2,521 (from 1,749 households) in the 2004 survey and 2,813 children (from 1,828 households) in the 2005 survey. Three hundred and sixty three households were involved in both surveys (19.9% of 2005 households).

### Characteristics of the study sample

The median number of children per EA was 29 in 2004 (range 11-51) and 31 in 2005 (range 17-54). District level characteristics of the enrolled population in the pre- and post intervention surveys are presented in Table 1. Both child and household level characteristics, overall, and by district, were comparable between surveys, with the exception that there were more households with more than 1 child in 2005 compared to 2004, and that caretaker education level was lower in Tone as compared to the other two districts. In 2005, 22.7% of enrolled children were below 18 months of age (i.e. an age group not targeted by the child health campaign).

**Table 1:** Characteristics of 5334 children who participated in the 2004 and 2005 surveys

	Pooled		Southern Coastal plain (Yoto)		Central Plateaux (Ogou)		Northern Savannah (Tone)	
	**2004**	**2005**	**2004**^**1**^	**2005**	**2004**	**2005**	**2004**	**2005**

**Child level(n)**	2521	2813	718	998	798	893	942	922
Age in month, mean(95%CI)	28.5 (27.8-29.1)	28.7 (28.1-29.4)	28.6 (27.4-30.1)	28.9 (27.6-30.2)	28.3 (27.2-29.3)	28.7 (27.5-30.0)	28.5 (27.4-29.6)	28.5 (27.7-29.3)
Male sex (%)	52.8	51.2	52.8*	48.8*	49.7	51.8	55.5	53.1
Sibling(s) <5 yr (%)	58.1*	63.3*	56.8	63.3	56.3	60.1	60.7	66.5
								
**HH level (n)**	1704	1828	495	648	564	594	645	586
# children (column %)								
1	59.8	56.4	61.4	56.5	61.3	59.9	57.4	52.7
2	36.7	36.1	33.9	35.8	36.3	32.5	39.2	39.9
>2	3.4	7.5	4.8	7.7	2.3	9.5	3.4	7.4
Caretaker (column %)								
Mother	89.7	88.9*	86.4	88.7	87.1	84.2	94.6	94.0
Father	6.0	3.7	6.5	2.3	9.2	6.2	2.9	2.6
Other	4.3	7.0	7.1	9.0	3.7	9.6	2.5	3.4
CT schooling (column %)								
None	73.3	76.6	62.6	70.4	66.8	68.2	87.1	92.0
Primary	20.6	18.3	27.8	24.4	26.1	23.6	10.4	6.1
≥secondary	6.0	5.1	9.6	5.2	7.1	8.2	2.5	1.9
SES rank score (%)								
<median^2^	50.1	51.1	46.1	42.7	53.7	57.4	50.1	53.9
% wealthiest Q			30.9	22.8	23.4	28.7	11.3	20.8
% poorest Q			16.4	19.5	20.9	15.4	18.5	13.4

HH = household; SES = Socio-economic Status, Q = quintile. *P < 0.05 2004 vs 2005, ** P < 0.01. ^1 ^Weighted analysis of Yoto 2004 data to take into account the variability of the enumeration area cluster size during the first 3 survey days (Crude number of children = 781). ^2 ^Median weighted socio-economic status calculated across district by survey

### Pre- and post-intervention rainfall patterns

Monthly district level rainfall patterns were compared for the pre- and post-campaign year (Figure 1). While rainfall patterns differ considerably between the three districts, similar rainfall patterns were observed within each of the 3 districts during 2004 and 2005.

### Bed net ownership and use

**Figure 2:**
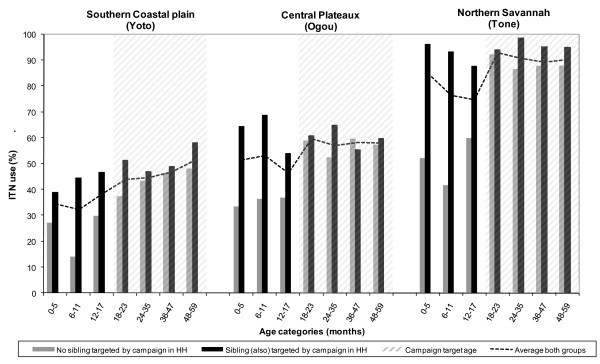
**2005, current ITN use by age and presence of a targeted sibling <5 years of age in the household**. Current ITN use was defined as reported ITN use during the previous night with a hanging ITN in the household.

Before the campaign nets of any kind were reportedly present in 5% to 12.7% of the households in the three districts (Table 2). Less than 1% of children were reported to have slept under an ITN the previous night. Nine months after the campaign, net ownership and use had increased dramatically, with over 60% of the enrolled households reported to own an ITN in all three districts (Table 2). Households in Tone that owned a net were more likely to hang their net than households in Ogou or Yoto (95.4% versus 75.7% and 62.9%, respectively, p < 0.001). Young children <5 years of age were present in 80.0%, 73.3% and 72.3% of eligible households in Yoto, Ogou and Tone, respectively. Based on the coverage survey results the community household level coverage of hanging ITNs (thus including households without young children) at the time of the survey was estimated to be 34.5%, 44.1% and 67.2% in Yoto, Ogou and Tone, respectively. ITN use during the previous night in the pooled group of children aged 0 - 59 months of age varied considerably between the three districts in the post-campaign survey, with prevalences of 35.9%, 43.8% and 80.6% in Yoto, Ogou and Tone, respectively (Table 2). As the campaign only targeted children between nine to 59 months, and the post-intervention survey occurred nine months post-campaign, ITN use was also assessed by age group (Table 2) and presence of a sibling who had been eligible for the campaign (Figure 2). The use of an ITN was generally shared with other household members. Only 1.2% of children reportedly slept alone under an ITN. In 91.8% the mother reportedly slept with the child under the ITN, and 35% of children reportedly shared with both their mother and one or more siblings.

**Table 2:** Reported and observed net coverage during the 2004 and 2005 surveys by district.

	**Pooled**^**a**^		**Southern Coastal plain**^**a **^ (Yoto)		Central Plateaux (Ogou)		Northern Savannah (Tone)	
	2004	2005	2004	2005	2004	2005	2004	2005
**Community level HH estimate **^**b**^								
N	2250	2402	750	789	750	808	750	805
Own ITN, %	0.03	59.7***	0.4	55.2	0.3	58.6	0.3	70.1
Hung ITN, %	-	45.7***	-	34.5	-	44.1	-	67.2
								
**Target group Household level**								
N	1749	1791	502	648	567	594	645	586
Own any net, %	9.5**	76.5***	11.6	67.4	12.7	75.0	5.0	87.9
Own ITN, % ^c^	0.4	73.6***	0.6	65.6	0.4	69.8	0.3	86.3
Hung ITN, % ^d^	-	58.3***	-	43.1	-	52.8	-	82.3
Hung >1 ITN, % ^d^	-	19.3***	-	6.9	-	12.1	-	40.0
								
# ITNs per household^e^								
>1 ITN, HHs 2 children, n(%)	-	200 (57.5)***	-	50 (46.3)	-	46 (46.5)	-	14 (73.8)
>1 ITN, HHs >2 children, n(%)	-	51 (81.0)	-	16 (80.0)	-	14 (63.3)	-	21 (100)
								
**Child net use previous night**								
N	2521	2813	781	998	798	893	942	922
Rep Any net use, %	6.2**	71.4***	7.5	59.1	9.9	66.3	2.1	89.7
Rep ITN use, %^f^	0.4	69.2***	0.7	57.1	0.3	62.7	0.2	88.6
Age <18mo, no target	0.2	59.7	0.5	45.7	0	55.5	0	78.8
Age ≥18mo, target	0.5	74.0	0.7	63.0	0.4	66.2	0.3	93.4
Rep ITN use + hanging, %^g^	-	53.2***	-	35.9	-	43.8	-	80.6
Age <18mo, no target		49.3***		27.5		38.7		70.1
Age ≥18mo, target		60.2***		40.3		46.4		85.7
ITN use by SES								
Wealthiest quintile, %	-	45.7***	-	36.3	-	34.2	-	76.3
Poorest quintile, %	-	52.7***	-	36.6	-	44.7	-	80.2

HH = Household, ITN = Insecticide Treated Nets; SES = Socio-Economic Status; Rep = reported; mo = months

^a ^Weighted analysis of 2004 Yoto data takes into account the variability of the enumeration area cluster size during the first 3 survey days (Crude number of households = 543); ^b ^These estimates are based on the household coverage survey that visited all selected households independent of the presence of children; ^c ^2004 data reported by caretaker, 2005 data based on direct observation at home visit (n households = 1791, n children = 2751 of 2813); ^d ^2004 data not collected, 2005 data based on direct observation; ^e ^Based on reported number of campaign nets received by households with >=2 children targeted by the campaign, i.e. >=18mo of age at time of 2005 survey (Yoto n = 128, Ogou n = 121, Tone n = 162); ^f ^"ITN use" was defined as a positive answer at the time of the morbidity survey in a central location to the question 'did this person sleep under an ITN last night?'; ^g ^"ITN hanging" was defined as a positive answer at during the home visit from the GPS team to the question whether a net was hanging. At this time, where people allowed, staff visually confirmed that an ITN was tied over a sleeping space, even if the net was folded above the bed. ** Differences between districts P < 0.001; *** Differences between districts = p < 0.0001.

### Reported history of malaria and anaemia and related treatment

There was a marked reduction in reported fever cases and health care attendance in Ogou (Table 3), but not in the other two districts. In Yoto, there was a smaller, but significant increase in reported fever cases for which health care was sought. The reported history of febrile episodes for which health care was sought, and the subsequent reported use of an anti-malarial or iron showed no decrease in 2005 compared to 2004 (Table 3).

**Table 3:** Reported history, health care seeking behavior, malaria and anaemia treatment in 2004 versus 2005 by district in 5331 children aged less than 5 years.

	Southern Coastal plain (Yoto)			Central Plateaux (Ogou)			Northern Savannah (Tone)		
	**2004** ** %**	**2005 ** **%**	**RR (95%CI)**	**2004** ** %**	**2005** ** %**	**RR (95%CI)**	**2004** ** %**	**2005** ** %**	**RR (95%CI)**

**History previous 2 weeks**^**1**^									
Febrile + HC sought^2^	13.1	21.7	1.66 (1.05-2.62)	26.6	8.7	0.33 (0.20-0.53)	28.0	23.6	0.84 (0.71-1.01)
Took antimalarials	34.0	30.9	0.91 (0.63-1.31)	39.4	34.3	0.87 (0.63-1.20)	44.7	40.0	0.89 (0.69-1.16)
Took iron	3.3	9.5	2.87 (1.36-6.08)	2.5	1.7	0.68 (0.29-1.58)	3.7	2.6	0.69 (0.30-1.61)
									
**History of severe illness in previous 2 months**									
Hospital admitted^3^	2.6	2.2		0.6	2.1		3.1	1.8	
Blood transfusion^4^	1.2	0.1		0.3	0.1		0.5	0.2	

RR = relative risk; CI = confidence interval

^1 ^The prevalence and RR were estimated using a binomial regression analysis with a log-link function, taking into account clustering at village level and adjusting for the age of the child, the socio-economic status of the household, and the education level of the caretaker. ^2 ^HC = Health care. ^3 ^Rao-Scott Chi-square test, taking into account clustering at village level. ^4^ The study was not powered to test for differences in these small numbers.

### Malaria morbidity

Overall, the prevalence of moderate-to-severe anaemia was 28% lower in 2005 compared to 2004 (PR = 0.72, 95%CI 0.62-0.84, adjusted for age group, SES and district) and there was a 0.35 g/dL (95%CI 0.25-0.45) increase in mean haemoglobin between the two survey points. Importantly, these differences between surveys depended on a combination of the district and the age category involved, in particular whether children were targeted by the campaign and were ≥18 months old or not at the time of the 2005 survey. When adding a three-way interaction term, survey × district × age group, to the model, its *P-*value was 0.048 for moderate to severe anaemia and 0.0008 for mean haemoglobin level. Because of this observed effect modification, results were also analysed stratified by both district and age group, as discussed below.

**Figure 3:**
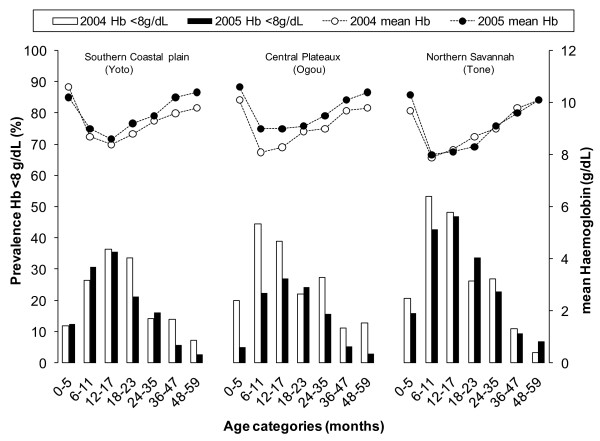
**Crude prevalence of moderate to severe anaemia and mean Haemoglobin (Hb) levels in the 3 districts, presented by age group and survey, taking into account clustering at village level**.

**Figure 4:**
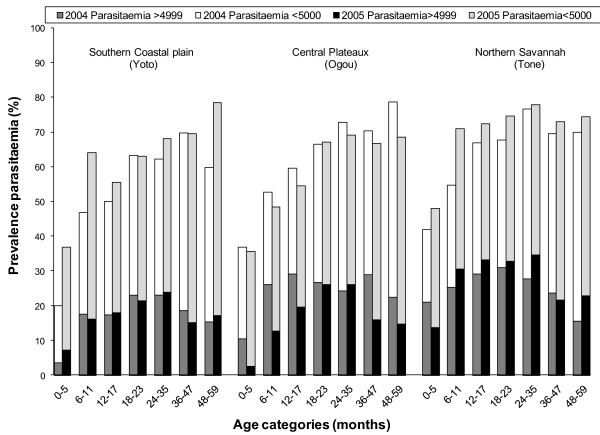
**Crude prevalence of parasitaemia in the 3 districts, presented by age group and survey for any parasitaemia and parasitaemia ≥5000/μL**.

**Figure 5:**
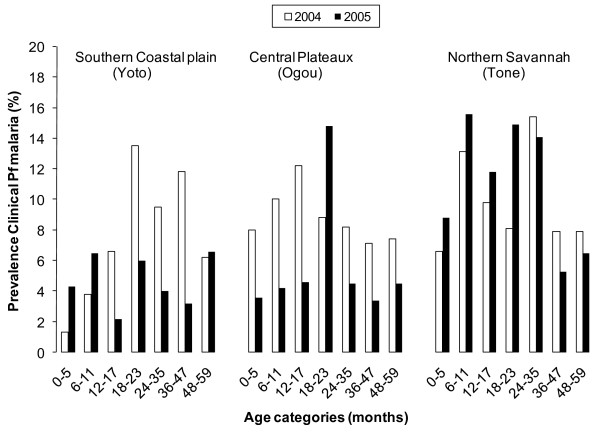
**Crude prevalence of *P*. *falciparum *(Pf) clinical malaria in the 3 districts, presented by age and survey**.

In the central district of Ogou, with the long, stable rainy season, and 43% of the children reportedly sleeping under ITNs in 2005, substantial differences were observed across most of the anaemia and malaria indicators. Comparable reductions among both children below and over 18 months of age (Table 4, Figure 3, Figure 4 and Figure 5), resulted in a pooled 50% and 42% reduction in prevalence of moderate to severe anaemia and clinical malaria, respectively, in children below five years of age with an increase of 0.6 g/dL in mean haemoglobin level. Little or no change was observed in parasite infection levels.

**Table 4:** Multivariate morbidity endpoints in 2004 versus 2005 by district and age group targeted by the campaign in 5331 children aged less than 5 years.^1^

	Age	Southern Coastal plain (Yoto)			Central Plateaux (Ogou)			Northern Savannah (Tone)		
	mo	2004 %	2005 %	RR (95%CI)	2004 %	2005 %	RR (95%CI)	2004 %	2005 %	RR (95%CI)
Hb <8 g/dL	<18	23.9	24.3	1.01 (0.73-1.41)	35.0	16.2	0.46 (0.33-0.64)	38.8	31.7	0.82 (0.65-1.02)
	≥18	15.2	9.4	0.62 (0.44-0.88)	17.4	9.4	0.54 (0.37-0.79)	14.5	15.7	1.08 (0.78-1.49)
Malarial anaemia	<18	15.0	19.8	1.31 (0.86-2.01)	23.2	8.6	0.37 (0.24-0.58)	28.6	28.9	1.01 (0.75-1.37)
	≥18	10.8	7.7	0.71 (0.48-1.05)	13.6	7.4	0.54 (0.33-0.90)	9.7	12.8	1.32 (0.89-1.95)
Clinical malaria	<18	5.9	3.4	0.59 (0.27-1.27)	10.0	3.9	0.38 (0.19-0.76)	11.4	10.4	0.91 (0.62-1.34)
	≥18	9.5	4.9	0.51 (0.35-0.75)	8.9	7.3	0.83 (0.54-1.28)	10.7	9.4	0.89 (0.59-1.33)
Any parasitaemia	<18	39.6	50.4	1.27 (1.02-1.59)	49.1	44.1	0.90 (0.75-1.07)	39.4	49.0	1.24 (1.05-1.47)
	≥18	64.1	71.3	1.11 (1.00-1.24)	71.1	68.1	0.96 (0.83-1.11)	70.7	74.5	1.05 (0.94-1.19)
High parasitaemia	<18	32.2	24.8	0.77 (0.48-1.24)	45.5	22.2	0.49 (0.33-0.71)	43.2	40.1	0.93 (0.67-1.28)
	≥18	30.4	26.4	0.87 (0.68-1.11)	36.0	28.0	0.78 (0.58-1.05)	31.5	36.2	1.15 (0.90-1.47)

RR = relative risk; CI = Confidence Interval; Hb = haemoglobin; mo = month; Clinical malaria = documented axillary temperature ≥37.5°C in the presence of malaria parasitaemia based on the slide result.; Malarial anaemia = Any parasitaemia + Hb < 8 g/dL; High parasitaemia = Parsitaemia >4999/μL.

^1 ^The prevalence and RR were estimated using a binomial regression analysis with a log-link function, taking into account clustering at village level and adjusting for the age (in categories 0-2,3-5,6-11,12-17,18-23,24-35,36-47,48-59) and sex of the child, the socio-economic status of the household (in quintiles), and the education level of the caretaker (no, primary, ≥secondary school).

In the southern district of Yoto in the coastal plain, with the lowest reported rate of current ITN use and community ITN coverage of the three evaluated districts, significant differences were only observed in the age group directly targeted by the campaign, i.e. children from the age of 18 months onwards (Figures 3, 4 and 5 and Table 4). While moderate to severe anaemia and clinical malaria were reduced by an average 34% and 50%, respectively in this group in 2005, no significant differences were seen in the younger age category (Figure 3, Figure 4, Figure 5 and Table 4). Similarly, a significant increase of mean haemoglobin of 0.50 g/dL, 95%CI 0.34-0.66, Figure 3) was only found in the targeted age group.

In the third, most northern district of Tone with its semi-arid climate and short rainy season, where over 80% of children were currently using ITNs and 40% of households had more than one campaign net hanging, no significant differences were observed in any age group. The younger age group up to 18 months showed a borderline significant reduction in moderate to severe anaemia prevalence (PR = 0.80, 95%CI 0.64-1.01), but this was not accompanied by a change in mean haemoglobin, or a difference in the prevalence of the assessed malaria markers (Figures 3, 4, 5 and Table 4).

### Malaria morbidity in ITN users versus non-users in 2005

In a weighted analysis of the 2005 survey, children who were reported to sleep under the ITN and had an ITN hanging in their household (observed) had significantly less moderate to severe anaemia compared to other children who either did not sleep under a net or did not have an ITN hanging in their household (PR = 0.81, 95%CI 0.67-0.98, adjusted for age and district). SES and sex were not found to be confounders. While ITN use or lack thereof during the previous night does not necessary relate well to persistent use, further analysis at district level suggested a trend where the difference between ITN users and non-users with respect to moderate to severe anaemia was biggest in the district with the lowest community coverage level, and smallest in the district with the highest community coverage level (Yoto PR = 0.70, 95%CI 0.48-1.03, Ogou PR = 0.86, 95%CI 0.61-1.22 and Tone PR = 0.89 (0.65-1.21). Similarly, a difference between these same groups was also found in mean haemoglobin; ITNs users had higher mean Hb than nonusers: 0.17 g/dL, 95% CI 0.03-0.31, adjusted for age and district). There was no effect modification by district and/or age).

## Discussion

The burden of malaria and anaemia in children <5 years was evaluated in three districts during two consecutive peak malaria transmission seasons before and after the 2004 Togo Integrated Child Health campaign. ITN ownership coverage reached 65% in 2005 in all three evaluated districts among households with children below the age of five years. This coincided with substantial reductions in moderate to severe anaemia and clinical malaria. Observed differences varied by region, and occurred mainly in the two districts from the southern coastal plain Maritime Region and hilly central Plateaux Region. The benefit was mainly observed in the age group of children ≥18 months who had been targeted by the campaign. The benefit was less clear in children <18 months. These children had not been targeted by the campaign themselves, but a considerable proportion shared a net with an older targeted sibling. No differences were observed in the northern district Tone in the semi-arid Savanes Region.

Clearly, the cross-sectional design with a historical control group limits the interpretation of these finding and factors other than ITNs could have contributed to the observed effect. For example, the package of child health interventions included mebendazole, which is known to have a beneficial impact on anaemia. Furthermore, some of the children (approximately 20%) in 2005 had also participated in the 2004 survey when they would have received iron and anti-malarial treatment if they were found to be anaemic or to have malaria. This may have provided some residual benefits one year later. The effect however, if any, would likely have been small. Seasonal differences between surveys may also explain the observed effect, however, all three sites experienced average and comparable rainfall in 2004 and 2005 with respect to the duration and timing of the rains. Moreover, the prevalence of moderate to severe anaemia in 2005 was 19% lower in ITN-users when compared to non-users. It seems therefore reasonable to attribute a significant proportion of the observed differences to the rapid scale-up of ITN coverage that took place between surveys rather than to natural differences in malaria exposure and transmission. Even though the dynamics between the intensity of transmission and weather are complex, the largest differences were observed in the central district with the longest and most stable rainy season of the three sites. This setting was most suitable for a cross-sectional comparison as the malaria transmission pattern in this area was relatively stable, and less sensitive to the exact timing of the survey.

It is not clear why the effect was observed in the southern two districts, but not in the northern district of Tone. ITN coverage was significantly higher in Tone than the two southern districts. In addition, if an ITN was present in the house, it was almost always hung, while in the southern areas nets were less likely to be observed hanging even though present in the house. This reflected that in Tone, a keep-up campaign was organized by the Red Cross which consisted of door-to-door home visits of Red Cross volunteers to explain the rationale and correct use of ITNs. Togo's most northern district has a much dryer semi-arid climate with a short rainy season and thus rapidly changing malaria transmission dynamics, making it more vulnerable to the potential confounding effects of season than the other sites. However, the rainfall patterns were almost identical in 2004 and 2005 and there was a lot of malaria in both years, and this is thus unlikely to explain the lack of effect of ITNs. Neither was there less malaria in Tone than in the other districts. Out of the three assessed districts, Tone was the only district with an active Lymphatic Filariasis Mass Drug Administration program. It is possible, of course, that other factors may explain the smaller effect of ITNs in the northern area of Togo than in the central and southern regions, such as a relative increase of nutritional deficiencies in Tone but not in the central and southern regions.

Despite the remaining questions about findings in Tone, these findings broadly confirm the known strengths and weaknesses of the current malaria burden indicators. Prevalence of moderate to severe anaemia, expected to reduce within 12 month of achieving high intervention coverage in malaria endemic areas, showed substantial changes in the range of those seen in RCT settings [13]. Parasitaemia prevalence, on the other hand, known to be relatively resistance to change in areas of high transmission [1], did not change or even increased. This probably indicates that the 9-months of intervention period was too short to pick up a change. Natural clearance of chronic asymptomatic infections can take time, and a substantial reduction in exposure to new infections may still amount to several new infections per month.

Despite their design limitations, national cross-sectional cluster-surveys play an important role in monitoring and evaluation of the impact of the increase in global malaria control efforts. The RBM MERG partnership has developed the Malaria Indicator Survey tool (MIS) [18], a national household morbidity survey that follows a standard two-stage cluster sampling design, and measures national level coverage in various interventions like intermittent preventive treatment (IPT), artemisinin combination therapy (ACT) and LLINs as well as impact indicators which have recently been conducted in a range of countries, including Zambia and Kenya [19]. These malaria specific surveys are planned at three- to four-year intervals and are designed to be conducted in conjunction with other monitoring activities that measure changes in all-cause childhood mortality, such as Demographic Health surveys (DHS) and Multiple Indicator Cluster Surveys (MICS). Each of these individual tools has its own limitations [5] but when used combined, provide a valuable and practical approach to assess the impact of malaria control efforts over time, particularly if they all show similar trends.

The Togo impact assessment, while a national cross-sectional survey using internationally recommended indicators and cluster sampling, was not a MIS survey. The main objective of the survey in Togo was to evaluate the short-term impact and effectiveness of the campaign as a delivery strategy, not to provide national or regional burden estimates in under 5 year olds. This survey had a limited budget (~$75,000 per survey rather than ~$1,000,000 of national MIS surveys), and was designed to capture some of the potential regional and age related variability in impact across the country. The observed complexity to burden changes at sub-national level is not captured by the current 'gold standard' MIS surveys and has not received much attention. As scaling-up of malaria control efforts is successful and reductions in the burden of malaria become apparent however, the focus of national malaria control programmes and their need for accurate estimates of progress soon requires more detail at sub-national level. Specifically, complementary M&E tools to the MIS surveys will be needed to capture the sub-populations and regions where control efforts need further strengthening, and guide national programmatic efforts, particularly now that countries are reconsidering their options for local malaria elimination. The assessment in Togo highlights both impressive progress, as well as the consequence of excluding young infants with this delivery strategy without additional keep-up or catch up distribution, even though a proportion of <18mo olds were reached by the campaign because they shared an ITN with a targeted sibling. The findings support subsequent adaptations of integrated campaigns, e.g. an integrated campaign in Tanzania in 2006 offered different intervention packages to different age groups [20] and universal distribution campaigns, and highlight the need to supplement integrated health campaigns with ITN distribution through ANCs or EPI contacts to achieve and maintain high coverage [21].

These findings add to the increasing number of reports that show the potential contribution of the use of integrated ITN distribution campaigns to reduce malaria morbidity in children. With this impressive scale-up of LLIN coverage and the switch of the first-line treatment policy to ACT, the Togo MoH is well underway to make significant progress in their efforts to control malaria in Togo.

## Competing interests

The authors declare that they have no competing interests.

Disclaimer: The findings and conclusions in this report are those of the authors and do not necessarily represent the views of the Centers for Disease Control and Prevention.

## Authors' contributions

DJT designed and coordinated the study, performed the statistical analyses and wrote the first draft of the paper. KM, AD1, AD2 and YS participated in the coordination of the study. AW, MJE and JvE supported the GPS component and participated in the coordination of the study. FtK and WAH participated in its design and participated in drafting the manuscript. All authors read and approved the final manuscript.

## Acknowledgements

We are grateful to the parents and guardians of the children who participated in the survey and the many field staff members and other people who assisted with and contributed to this project. This survey has been a joint international effort that has received support from various individuals and institutes. This study was financially supported by the Canadian Red Cross. We thank Poutougnima Tchamdja, Directeur Général de la Santé for the support from the Togo Ministry of Health. We are grateful to Mr Norbert Paniah, Antoinette Awaga, Blaise Edoh and Messan Nyonato from the Togolese Red Cross for their logistical support. At the International Federation of the Red Cross, we thank Jean Roy and for his assistance. We also express our gratitude to Sanofi-Synthelabo in France and DAFRA in Belgium for their kind donations of Arsucam, the anti-malarial drug used in this survey. Similarly we thank Part Peeters, Chris Weeks and their colleagues at DHL for their expert assistance with the transportation of all involved study shipments for the first survey. At LSTM we thank Philip Gichuru for his statistical support.
